# The Translational Regulation in mTOR Pathway

**DOI:** 10.3390/biom12060802

**Published:** 2022-06-08

**Authors:** Miaomiao Yang, Yanming Lu, Weilan Piao, Hua Jin

**Affiliations:** Key Laboratory of Molecular Medicine and Biotherapy, School of Life Sciences, Beijing Institute of Technology, No. 5 South Zhongguancun Street, Beijing 100081, China; 3120191380@bit.edu.cn (M.Y.); 3120211425@bit.edu.cn (Y.L.)

**Keywords:** mTOR, translational regulation, 4E-BP, S6K, LARP1

## Abstract

The mechanistic/mammalian target of rapamycin (mTOR) plays a master role in cell proliferation and growth in response to insulin, amino acids, energy levels, and oxygen. mTOR can coordinate upstream signals with downstream effectors, including transcriptional and translational apparatuses to regulate fundamental cellular processes such as energy utilization, protein synthesis, autophagy, cell growth, and proliferation. Of the above, protein synthesis is highly energy-consuming; thus, mRNA translation is under the tight and immediate control of mTOR signaling. The translational regulation driven by mTOR signaling mainly relies on eukaryotic translation initiation factor 4E (eIF4E)-binding protein (4E-BP), ribosomal protein S6 kinase (S6K), and its downstream players, which are significant in rapid cellular response to environmental change. mTOR signaling not only controls the general mRNA translation, but preferential mRNA translation as well. This means that mTOR signaling shows the stronger selectivity to particular target mRNAs. Some evidence has supported the contribution of 4E-BP and La-related proteins 1 (LARP1) to such translational regulation. In this review, we summarize the mTOR pathway and mainly focus on mTOR-mediated mRNA translational regulation. We introduce the major components of mTOR signaling and their functions in translational control in a general or particular manner, and describe how the specificity of regulation is coordinated. Furthermore, we summarize recent research progress and propose additional ideas for reference. Because the mTOR pathway is on the center of cell growth and metabolism, comprehensively understanding this pathway will contribute to the therapy of related diseases, including cancers, type 2 diabetes, obesity, and neurodegeneration.

## 1. mTOR Pathway

The mechanistic/mammalian target of rapamycin (mTOR), an evolutionarily conserved serine/threonine kinase, is an important member of the phosphoinositide 3-kinase-related kinase (PIKK) family [1]. The target of rapamycin (TOR) was first discovered in 1991 during the screening rapamycin-resistant yeast mutants as a target of the immunosuppressant and anticancer drug rapamycin. Shortly thereafter, in 1994, its mammalian homologue mTOR was discovered through several independent studies [2,3,4,5,6,7]. mTOR is also known as FKBP-rapamycin-associated protein (FRAP) or rapamycin FKBP12 target (RAFT1). Previous studies on the mechanism of rapamycin in cell cycle arrest had shown that rapamycin binds to FK506 binding proteins (FKBPs), mainly to FKBP12. Later research found that the main protein targeted by FKBP12-rapamycin complex was kinase mTOR [3,5,6,7,8]. Therefore, rapamycin is commonly used as an mTOR inhibitor.

mTOR regulates cell proliferation and growth rate by responding to various stimulation including hormones (insulin), growth factors (like insulin-like growth factor (IGF)), nutrients (amino acids), energy levels, and oxygen content [9,10,11]. In order to grow and divide, cells must increase anabolism, including the synthesis of proteins, lipids, and nucleic acids, and meanwhile, inhibit catabolism such as protein turnover via suppressing both autophagy and ubiquitin-proteasome system (UPS). mTOR relays proliferative and anabolic signals to downstream transcriptional and translational apparatus thereby regulating the energy production, protein synthesis, autophagy, and cell cycle in G1/S and G2/M phase [1,4,11,12,13,14,15,16].

Protein synthesis consumes a large portion of cellular energy; thus this process is usually associated with energy levels in cells. Previous studies have shown that mTOR is an essential regulator of mitochondrial energy production and appears to promote mitochondrial biogenesis and oxidative metabolism to enhance energy production [9,10,17,18,19]. mTOR also plays a role in repressing autophagy, thus inhibiting protein catabolism. During autophagy, unrequired or dysfunctional components are removed and recycled through a lysosome-dependent mechanism. Autophagy is inhibited by mTOR through two approaches. The first is that mTOR directly inhibits unc-51-like kinase 1 (ULK1), the key factor in autophagy induction [20]. The second is that mTOR blocks lysosome biogenesis through inhibition of the nuclear translocation of transcription factor EB (TFEB) and thus indirectly inhibits autophagy [21,22,23]. The downregulated autophagy ensures sufficient cellular bio-macromolecules for proliferation.

Many mutations in the mTOR pathway leading to its hyperactivity have been identified in cancer [24]. Both loss of function mutations in the negative mTOR regulators such as phosphatase and tensin homolog (PTEN), tuberous sclerosis complex (TSC1/2), neurofibromatosis type 1 (NF1), liver kinase B1 (LKB1), also known as serine/threonine kinase 11 (STK11) and gain of function mutations in positive mTOR regulators such as AKT (also known as protein kinase B (PKB)) and phosphoinositide-3-kinase (PI3K) result in hyperactivity of mTOR signaling in a large number of malignant tumors [25,26]. All these demonstrate that mTOR plays a central role in the occurrence and progression of a variety of cancers. Therefore, so much attention has been devoted to understanding mTOR signaling pathway and to developing therapies that target this pathway [27].

### 1.1. Upstream Players of mTOR

**Figure 1:**
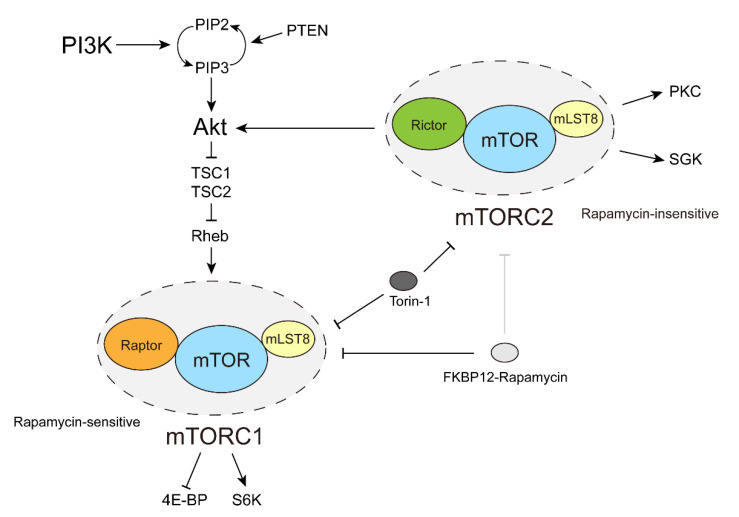
mTOR pathway. The major signaling pathways of mTORC1 and mTORC2. Arrows indicate facilitation and activation, and blocks indicate inhibition.

The mTOR upstream consists of varying growth factors and signaling molecules related to mitosis, such as PI3K, PDK1 (phosphoinositide-dependent kinase 1), TSC1-TSC2 complex, and Rheb, etc. Therefore, mTOR involves multiple life activities and links multiple signaling pathways, of which one of the most important is the PI3K-Akt-mTOR signaling pathway (Figure 1) [28]. PI3K-Akt-mTOR regulates organismal metabolism and determines cellular fate. It is associated with a variety of life activities, such as cellular glucose transport, cell proliferation, differentiation, apoptosis, dormancy, carcinogenesis, and longevity [11,29]. In diverse cancers, this pathway is dysregulated, so the components of this pathway are highly valued [24,30,31,32].

### 1.2. Two mTOR Complex

mTOR exists as the catalytic subunit of two different complexes, mTOR complex 1 (mTORC1) and mTOR complex 2 (mTORC2), which have different composition and function. mTORC1 includes three core components, mTOR, mammalian lethal with Sec13 protein 8 (mLST8) and regulatory protein associated with mTOR (Raptor). Similarly, mTORC2 also contains mTOR and mLST8, whereas there is no Raptor in mTORC2 but rapamycin-insensitive companion of mTOR (Rictor) instead [11,33]. The common subunit mLST8 in two complexes probably assists mTOR in forming the stable kinase activation loop, and Raptor and Rictor facilitate the recruitment of substrates to mTOR [34,35,36]. In addition to these core components in the complexes, two inhibitory subunits, proline-rich Akt substrate of 40 kDa (PRAS40) and DEP domain containing mTOR interacting protein (DEPTOR) are included in mTORC1 [37,38,39,40], and DEPTOR as well as the regulatory subunits Protor1/2 and mSin1 are involved in mTORC2 [11,38,41,42,43,44,45,46].

Given the importance of the mTOR pathway in various diseases, especially in most cancers, inhibitors targeting key players in the pathway have undergone therapeutic development. Rapamycin and its analogs (rapalogs) belong to the first generation mTOR inhibitors. Rapamycin is associated with the FRB domain of mTOR through another protein FKBP12 so it does not directly bind to the mTOR kinase domain. Still, in three-dimensional conformation, FKBP12-rapamycin blocks the catalytic hole in the mTOR kinase domain, thereby impairing entrance of substrates to the catalytic hole [47]. Rapamycin is known to have significant inhibitory effects on mTORC1 yet only marginal effects on mTORC2 [48,49]. It should be noted that rapamycin likely inhibits some of the mTORC1 substrates such as S6K more efficiently than others [25,27,50]. The second-generation inhibitors PP242, INK128, and Torin-1 belong to ATP analogs and target the kinase domain of mTOR by competing with ATP for binding, thus inhibiting both mTORC1 and mTORC2 [32,51,52,53]. In third-generation inhibitor RapaLINK, rapamycin and an ATP analog are chemically linked together, so RapaLINK has the performance of both rapamycin and ATP analog, reducing drug-resistant problems in mTOR mutated cancers [54].

Due to the lack of an mTORC2-specific inhibitor, less is known about mTORC2 than about mTORC1. What is established, however, is that growth factors such as insulin can activate mTORC2. Once activated, mTORC2 phosphorylates Ser473 of AKT1 and participates in cell apoptosis, glucose metabolism and other life activities through AKT. Additionally, mTORC2 can also bind to and regulate PKC (protein kinase C) that is involved in cytoskeleton reorganization. Another target protein of mTORC2 is SGK (serum- and glucocorticoid-induced protein kinase 1), which is important for ion transport [55].

### 1.3. Downstream Effectors of mTOR

**Figure 2:**
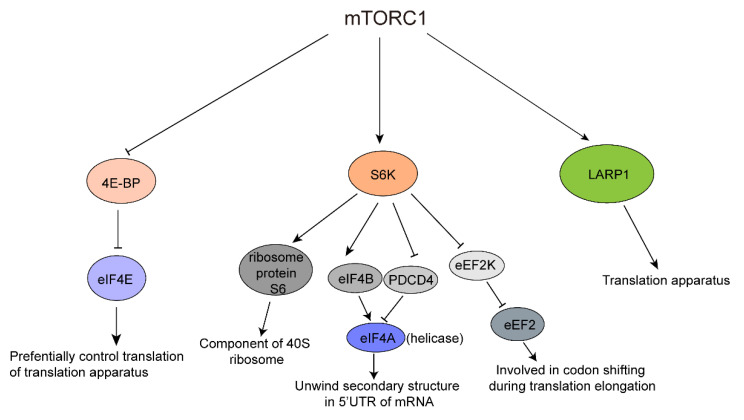
The main downstream signaling pathways of mTORC1. Arrows indicate facilitation and activation, and blocks indicate inhibition.

Activated mTORC1 phosphorylates a variety of downstream substrates (Figure 2). Major downstream substrates of mTORC1 include ribosomal protein S6 kinase (S6K) and eIF4E (eukaryotic translation initiation factor 4E)-binding protein (4E-BP), which act in parallel to control the translation of mRNA.

Only one homolog of S6K is expressed in *Drosophila*, whereas two homologs, S6K1 and S6K2, are expressed in mammals and mTORC1 mainly activates S6K1 [56,57]. Activated protein kinase S6K transmits upstream signals to various effectors for regulating diverse cellular processes, such as inhibiting glycogen synthase kinase-3 (GSK3) [58], Bcl-2/Bcl-XL-antagonist, causing cell death (BAD) [59] and mouse double minute 2 (Mdm2) [60] for cell survival, activating cAMP-response-element modulator τ (CREMτ) [61] and oestrogen receptor (ERα) [62] for transcription, enhancing the activity of S6K1 Aly/REF-like substrate (SKAR) [63,64] and Cap binding protein 80 (CBP80) [65] for RNA processing. Furthermore, activated S6K also suppresses programmed cell death protein4 (PDCD4) [66] and eukaryotic elongation factor-2 kinase (eEF2K) [67] and activates eukaryotic translation initiation factor 4B (eIF4B) [68,69] and ribosomal protein S6 (rpS6) for translation, and controls some unknown factors for cell motility, growth and size, and adipocyte differentiation [70] (Figure 2).

There are three homologs of 4E-BP in mammals: 4E-BP1, 4E-BP2, and 4E-BP3; only one in *Drosophila* named Thor; and two in *Saccharomyces cerevisiae*, Caf20p (also known as p20) and Eap1p [71]. 4E-BPs directly bind to the mRNA cap-binding protein eIF4E which is a component of the eukaryotic translation initiation complex (eIF4F). The eIF4F complex includes eIF4E, RNA helicase eIF4A, and scaffold protein eIF-4G. This complex recruits the small ribosomal subunit 40S to mRNA to start cap-dependent translation initiation. Actually, the eIF4F complex is considered to be a rate-limiting factor because the eIF4E protein level is believed to be tightly regulated. Phosphorylated and activated 4E-BP occupies the eIF4G binding region in eIF4E and represses the formation of translation initiation complex eIF4F, which ultimately represses the cap-dependent translation initiation and reduces most mRNA expression [72].

## 2. Translational Regulation by mTOR Pathway

**Figure 3:**
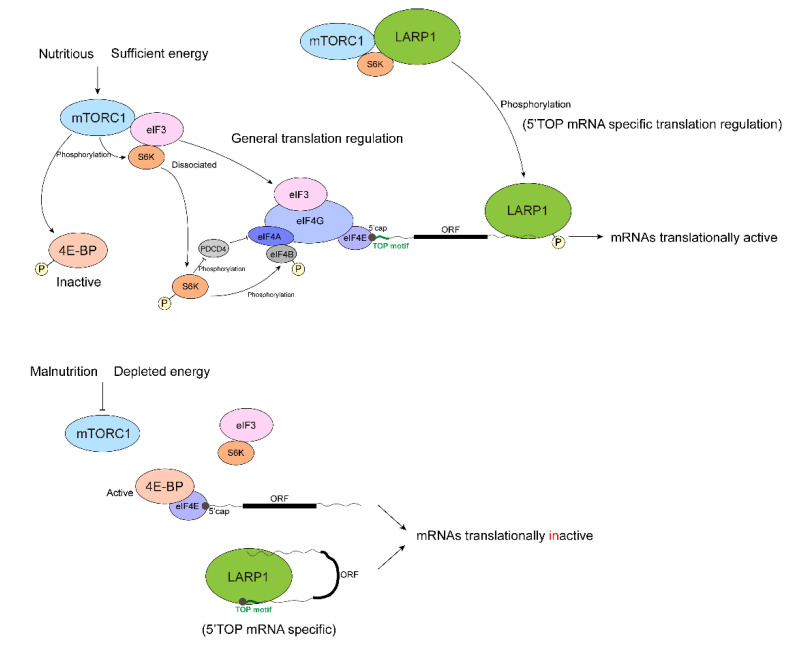
S6K and 4E-BP in mRNA translation. When mTORC1 is activated, 4E-BP is phosphorylated and inactive, and LARP1 is phosphorylated and binds to the 3′ UTRs of RP mRNAs, promoting their translation. When mTORC1 is inactivated, 4E-BP binds to eIF4E and inhibits eIF4F complex formation. S6K1 binds to eIF3 complex to block translation initiation. LARP1 directly binds to 5′ caps, 5′ TOP sequences, and 3′ UTRs of RP mRNAs and inhibits their translation.

Eukaryotic mRNA translation can be regulated at any process in initiation, elongation, and termination but is mainly regulated during initiation, allowing rapid, temporal, and spatial control on gene expression. Eukaryotic mature mRNA contains 5′ cap that is indispensable for cap-dependent translation initiation, the primary and canonical way for eukaryotes to initiate translation. S6K and 4E-BP in mTOR pathway can corporately participate in translation control through various ways (Figure 3).

### 2.1. General Translational Control by mTOR Pathway

Without signaling from mTOR, S6K1 binds to the eIF3 complex to block translation initiation. Activated mTORC1 phosphorylates and activates S6K1, dislocating eIF3 from S6K1 and acts as the translation initiation complex [70]. Although mTORC1 phosphorylates 4E-BP, leading to eIF4F complex formation. S6K1 further phosphorylates its substrates, like rpS6, eIF4B, PDCD4, and eEF2K, to activate translation [70,73]. RpS6 is a component of the 40S ribosome, being in close proximity to the 60S ribosome and translating mRNAs. Phosphorylated and activated eIF4B is recruited to helicase eIF4A, and assists its helicase activity in unwinding secondary structure in 5′ UTRs of mRNAs. Furthermore, S6K1 also enhances eIF4A activity via promoting degradation of PDCD4, the tumor suppressor and the inhibitor of eIF4A [74]. Phosphorylated and inactivated eEF2K does not inhibit eEF2, so eEF2 could be involved in ribosomal translocation during mRNA translation elongation. Thus, S6K targets several key factors in translation, ensuring precise regulation.

Remarkably, S6K may increase the translation of a set of mRNAs with long and structured 5′ UTRs via enhancing RNA helicase activity. Actually, the unwound structure of mRNA is essential for its translation, allowing the translation initiation complex to move along mRNA to locate the translation initiation codon. Importantly, many mRNAs with highly folded 5′ UTRs encode proteins crucial to biological activities, such as MyC, HIF1α, ODC1, cyclin D1, fibroblast growth factor (FGF), vEGF, and IGF, = and all of these contribute to efficient cell cycle progression [75].

### 2.2. Preferential Translational Control by mTOR Pathway

Previous studies have showed that almost all (99.8%) mRNAs exhibit translational inhibition after mTOR inhibitor torin-1 treatment in cells identified by ribosome profiling and translational efficiency decrease by 50% roughly [76,77]. It is worth noting that some mRNAs are affected more obviously, that is, they show a stronger selectivity to inhibit some mRNAs. Their functions are mainly related to protein synthesis, and many of them have TOP/TOP-like motif [32,76]. Torin-1 hardly affects those mRNA translations after double-knocking out 4E-BP1/2 [76], while rapamycin represses translational activity of them in S6K1 knockout or S6K1/2 double-knockout mice [78,79], indicating that the regulation of mTOR on those mRNA translation largely depends on 4E-BP. Although inhibiting the formation of eIF4F can explain the general cap-dependent reduction in translation rate, it cannot explain the preference for particular mRNA suppression. Actually, several researchers have shown that 4E-BP represses the translation of a subset of mRNAs [80].

Researchers have proposed two models about the mechanism of 4E-BP-mediated regulation preference. One is that 4E-BP only binds to eIF4E but is not associated with mRNAs, which inhibits the formation of rate-limiting factor eIF4F and eIF4E-dependent translation of mRNAs. The other model is that 4E-BP binds to eIF4E, which is further associated with mRNAs to inhibit the translation of bound mRNAs. Although these two models are not in conflict and can exist simultaneously, because of technical limitations, there is the strong in vitro evidence based on the pull-down assay using capped mRNA chromatography but no direct in vivo evidence proving the second model [81,82,83]. Fortunately, the development of the new technique “targets of RBPs identified by editing” (TRIBE) has provided in vivo evidence for the second model.

TRIBE fuses an RNA binding protein (RBP) to the catalytic domain of *Drosophila* RNA-editing enzyme adenosine deaminase RNA specific (ADAR) and expresses the fusion protein in vivo. When the RBP binds to its target RNAs, ADAR can convert a part of adenosines in the RNAs into inosines—that is, the target RNAs are edited. Importantly, the editing events are irreversible, marking the target RNAs permanently after the binding. And those RNAs with the labels are identified in high-throughput sequencing by the change of A-to-G [84]. Improved version HyperTRIBE possesses an amino acid substitution E488Q in ADAR, having increased the editing efficiency and reduced sequence bias [85]. HyperTRIBE in mammals uses the catalytic domain of human ADAR2 (hADAR2-E488Q) instead of fly ADAR [86,87,88].

By applying hyperTRIBE to 4E-BP, it has been confirmed that 4E-BP is associated with particular mRNAs especially when mTOR is inactivated by its inhibitors in both flies and mammals. 4E-BP is further confirmed to be in close proximity to RNA by cross linking and immunoprecipitation (CLIP) experiment in flies as well. And the targets of 4E-BP are highly enriched in the pathway of translation and immune response in both organisms. The targets in Drosophila include translational apparatus such as those encoding ribosome proteins (RPs), and most subunits of the eukaryotic translation initiation factor 3 (eIF3) [86]. Interestingly, some eIF3 subunits have been found to play an important role in the translational regulation of specific mRNAs [89,90]. Furthermore, PRTE (pyrimidine-rich) motif is enriched in the 5′ UTRs of 4E-BP targets, and the translation of mRNAs with this motif are strongly inhibited after mTOR inactivation in both organisms. Thus, 4E-BP not only generally inhibits eIF4E-cap-dependent translation, but preferentially regulates the translation of mRNA with PRTE motif in both flies and mammals as well [76,86]. To date, the mechanism of how 4E-BP preferentially selects its target mRNAs is still a mystery in flies and mammals. Whether it is derived from the activated 4E-BP itself, the eIF4E preference for 5′ terminal RNA sequences [91] or other factors such as RBPs is undetermined.

The translational control element comprised of oligo-pyrimidine, the sequence composition of PRTE, TOP-like, or pyrimidine-enriched sequence (PES) is similar to the well-known conserved motif 5′ terminal oligopyrimidine tract (5′ TOP) except for the more restricted localization of classical 5′ TOP to immediate downstream of transcription start sites (TSS). The classical TOP mRNAs have an invariable cytosine at 5′ terminal followed by 4–15 uninterrupted pyrimidines, encoding mostly translation factors and nearly all RPs [77,92,93]. Silico re-analysis using the TSS annotation obtained by HeliScopeCAGE has indicated that PRTE in mammalian mTOR targets is overrepresented around the TSS regions. This suggests 5′ TOP and PRTE may represent the same translational control element [32,94]; however, further verification is needed. Most genes generate multiple TSSs but only the main TSSs are annotated. Furthermore, for a given gene, some TSSs may have TOP but others may not. To predict the TOP element precisely even in the case of genes with heterogeneous and unannotated TSSs, a metric called TOPscore has been introduced [77]. The definition of TOPscore is that the average length of consecutive C/U at all positions in CAGE peaks within 1 kb of the annotated main TSS, weighted by the number of CAGE reads at each position [77]. TOPscore considers the important contributors to mTOR-driven translational regulation of TOP mRNAs including both TOP motif lengths and heterogeneous TSSs in the prediction, and provides a good metric by which to quantify sequence features and identify TOP mRNAs [77].

In addition to 4E-BP, La-related proteins 1 (LARP1) is reported as a major contributor to translational regulation in mTOR pathway. A large RBP, LARP1 consists of eIF4G-like motif, RNA-binding La motif (LAM) that is conserved in LARP superfamily, RNA recognition motif-like (RRM-L) domain, and its unique region DM15 in C terminus [77,93,95]. A series of reports have demonstrated the multiple functions of LARP1 in the control of TOP mRNA translation [77,95,96,97,98,99] and in mRNA stability [96,97,100,101], whereas the disruption of LARP1 decreases the growth rate in some tumors [102,103,104]. LARP1 has been found to be associated with 3′ terminus of the poly(A) tail and thereby stabilized mRNAs [97,100]. Also, polysome analysis indicated that LARP1 is associated with polysomes via PABP in an mTOR activity-dependent manner. In the mTOR active conditions, LARP1 enhances general mRNA translation and makes a more significant effect on TOP mRNA translation [98].

Recently, some solid evidence has emerged to support the observation that LARP1 is a major player in the translational regulation of TOP mRNAs driven by mTOR. It has been proposed that LARP1 is under control of mTORC1 via RAPTOR and S6K1, and functions as a repressor of TOP mRNA translation in mammals when mTOR is inactivated. This is carried out through direct binding to the 5′ cap and 5′ TOP regions of transcripts [96,97,99]. Polysome analysis, Ribo-seq, and in vitro translational assay utilizing LARP1-depleted cells with or without the treatment of mTOR inhibitors support that LARP1 represses TOP mRNA translation in the mTOR inactivated condition [77,95,96,97]. The X-ray crystal structure of the LARP1 DM15 region has indicated that this region recognizes the 7-methylguanosine cap and invariant 5′ cytidine of TOP mRNAs. The electrophoretic mobility shift assay (EMSA) also has shown that LARP1 binds to the capped 5′ TOP RNA fragments in vitro, thereby repressing translation. Moreover, LARP1 may also protect TOP mRNAs from de-capping factors [95,105]. Photoactivatable ribonucleoside-enhanced crosslinking and immunoprecipitation (PAR-CLIP) of LARP1 with or without the treatment of an mTOR inhibitor has revealed that in the mTOR inactivated condition, LARP1 will induce PES accumulation in the 3′ end of 5′ TOP-containing 5′ UTRs. In addition, LARP1 is able to bind to 3′ UTRs in both mTOR activated and inactivated conditions and still prefers binding to PES in 3′ UTRs [97]. It is worth mentioning that even if LARP1 binds to 5′ TOP including 5′ cap in vivo, the CLIP Seq-based methods barely detect CLIP signals in 5′ TOP regions due to 5′ capped RNAs being unable to be ligated to the 5′ linkers (adaptors) during sequencing library preparation [97,106]. It may be the reason why the canonical 5′ TOP regions have not been detected in PAR-CLIP.

Considering all these findings, a more acceptable model (Figure 3) has been proposed. When mTORC1 is activated, phosphorylated 4E-BP is silenced. LARP1 is phosphorylated by mTORC1 and Akt/S6K1, and binds to the 3′ UTRs of its target mRNAs including TOP mRNAs, promoting their translation [97,105]. When mTORC1 is inactivated, hypo-phosphorylated LARP1 directly binds to the 5′ caps, 5′ TOP sequences, and 3′ UTRs of TOP mRNAs to inhibit their translation (Figure 3). Simultaneously, the hypo-phosphorylated form of 4E-BP binds to eIF4E along with mRNA 5′ cap regions and inhibits the formation of eIF4F. Perhaps there is competition between 4E-BP- eIF4E and LARP1 for binding to 5′ TOP mRNAs at this moment, which may ensure the complete translational inhibition of TOP mRNAs.

## 3. Prospect

Protein synthesis is one of the most energy-consuming processes in cells. mTOR plays a central role in rapid adaption of cells between energy-rich and energy-depleted states by regulating mRNA translation. Regulation preference enables cells to shut down the synthesis of unnecessary proteins in energy-depleted conditions. Large numbers of translational apparatuses are mainly required for cell growth and proliferation, but not for cells in a quiescent state, so it is reasonable to repress preferentially their translation in a harsh condition and keep cells in a quiescent state. On the contrary, mRNA translation and mTOR pathways are often up-regulated in some abnormal proliferative diseases, including malignant tumors.

The translational regulation in mTOR pathway is an intricate web and only the key players 4E-BP, S6K, and LARP1 are discussed here. Although these factors can address the main phenomena observed in mTOR translational regulation, there are still some unsolved issues. Some mRNAs under mTOR translational control don’t have the TOP/PRTE motif and also not all TOP mRNAs are under control of 4E-BP, S6K, and LARP1. This raises the possibility that some unknown proteins may be involved in the pathway. In the past decade, RNA modifications such as m6A (N6-methyladenosine) have evolved as a new regulatory element of mRNA metabolism. As the most abundant internal modification in eukaryotic mRNAs, m6A modifications are reversible and recognized by effector proteins, thereby controlling the translation, stability, localization of the modified mRNAs [107,108,109,110,111]. Interestingly, mTORC1 is reported to enhance the synthesis of the major methyl donor S-adenosylmethionine (SAM) by controlling methionine adenosyltransferase 2 alpha (MAT2A) expression. mTORC1 also upregulates the level of Wilms’ tumor 1-associating protein (WTAP), the positive regulatory subunit of m6A RNA methyltransferase complex. Through stimulation of m6A RNA modifications, mTORC1 signaling promotes protein synthesis and cell growth [112]. It will be important to identify and elucidate new RBPs, cap-binding proteins, and RNA modification–related proteins in the mTOR pathway. The profound knowledge of mTOR signaling will be indispensable for the development of novel therapies of cancer and metabolic diseases.

## Data Availability

No applicable.
